# Initial experience of temperature-controlled irrigated radiofrequency ablation for ischaemic cardiomyopathy ventricular tachycardia ablation

**DOI:** 10.1007/s10840-022-01158-4

**Published:** 2022-02-22

**Authors:** Jaffar Al-Sheikhli, Ian Patchett, Ven Gee Lim, Leeann Marshall, Will Foster, Michael Kuehl, Shamil Yusuf, Sandeep Panikker, Kiran Patel, Faizel Osman, Prithwish Banerjee, Nicolas Lellouche, Tarvinder Dhanjal

**Affiliations:** 1 Department of Cardiology, University Hospital Coventry & Warwickshire NHS Trust, Coventry, UK; 2 University of Warwick, Coventry, UK; 3 Avenue du Marechal de Lattre de Tassigny, Hopital Henri Mondor Albert Chenevier, Creteil, Inserm U955, 94000 Paris, France

**Keywords:** Ventricular tachycardia, Ischaemic cardiomyopathy, Temperature-controlled irrigated radiofrequency ablation, Feasibility

## Abstract

**Background:**

The DiamondTemp ablation (DTA) catheter system delivers high power, open-irrigated, temperature-controlled radiofrequency (RF) ablation. This novel ablation system has not been previously used for ventricular tachycardia (VT) ablation.

**Objective:**

Feasibility of using the DTA catheter system for VT ablation in ischaemic cardiomyopathy (ICM) patients*.*

**Method:**

Ten ICM patients with optimal anti-arrhythmic drug therapy and implantable cardiac defibrillators (ICD) were recruited. VT inducibility testing was performed at the end of the procedure. ICD data for device detected VT episodes and device treated VT episodes were collected for 6-months pre- and post-ablation.

**Results:**

Substrate analysis demonstrated reductions in the borderzone area of 4.4 cm^2^ (*p* = 0.026) and late potential area of 3.5 cm^2^ (*p* = 0.0449) post-ablation, with reductions in the mean bipolar and unipolar voltages of the ablation target areas (0.14 mV (*p* = 0.0007); 0.59 mV (*p* = 0.0072) respectively). Complete procedural success was achieved in 9 procedures. Post-ablation VT inducibility testing was not performed in 1 procedure due to a steam pop complication resulting in pericardial tamponade requiring drainage. Mean follow-up of 214 ± 33 days revealed an 88% reduction in total VT episodes (*n* = 266 median 16 [IQR 3–57] to *n* = 33 median 0; *p* = 0.0164) and 77% reduction in ICD therapies (*n* = 128 median 5 [IQR 2–15] to *n* = 30 median 0; *p* = 0.0181).

**Conclusion:**

The DTA system resulted in adequate lesion characteristics with effective substrate modification, acute procedural success and improved outcomes at intermediate-term follow-up. Randomised controlled trials are required to compare the performance of the DTA system against conventional ablation catheters.

## Introduction

Current therapeutic approaches for ventricular tachycardia (VT) including invasive catheter ablation (CA) and anti-arrhythmic drug (AAD) therapy lack long-term efficacy and are associated with significant side effects [1]. The delivery of transmural and durable lesion creation remains challenging when using traditional focal irrigated radiofrequency (RF) ablation catheters. Treatment failure with CA can be due to the inability to deliver adequate ablative energy across ventricular myocardium as long-term outcome following CA remains variable, with high rates of VT recurrence [1–3]. Thus, there is an urgent need to develop novel CA technologies.

**Fig. 1:**
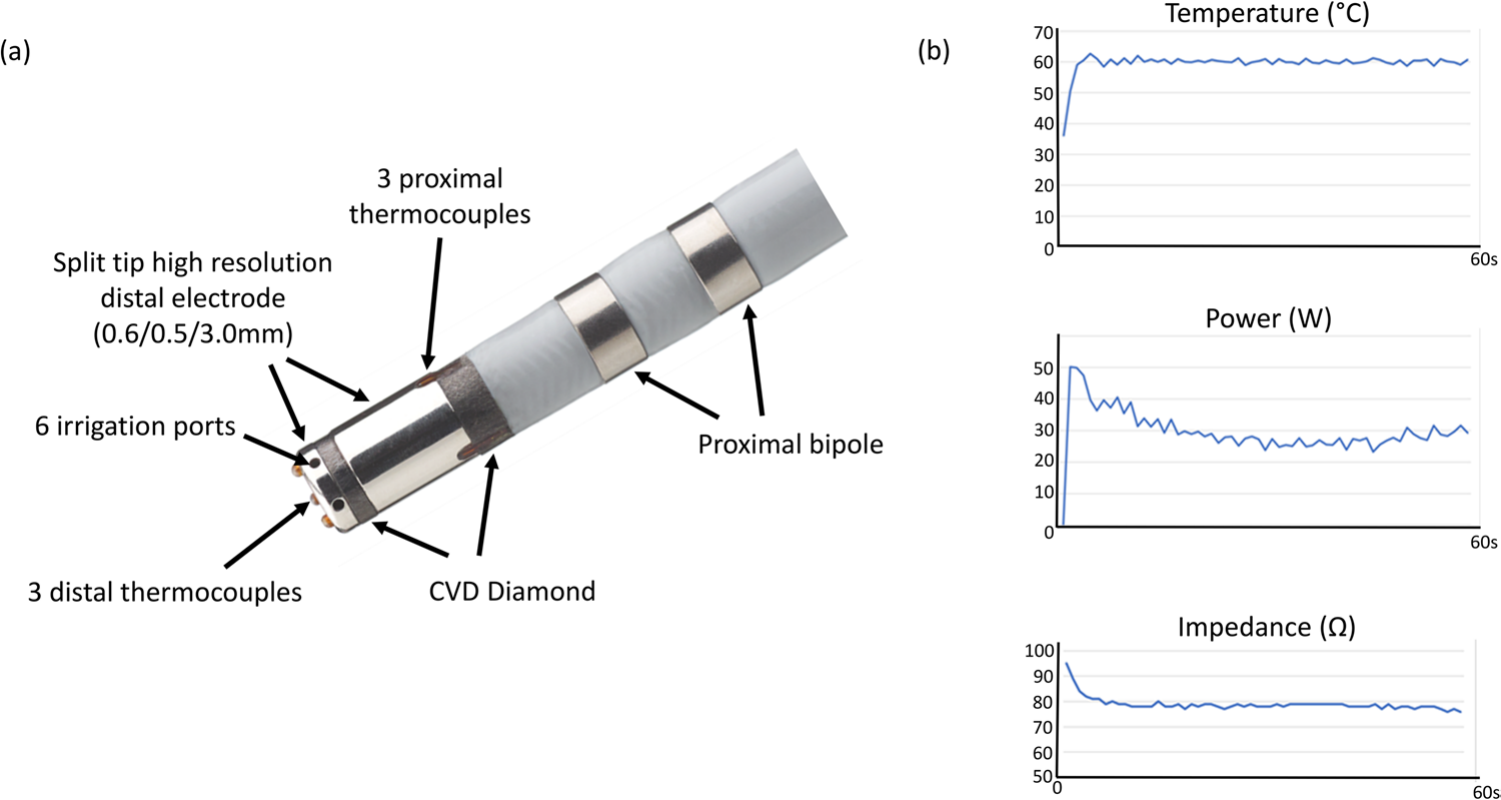
The DiamondTemp catheter. The DiamondTemp ablation (DTA) (Medtronic, Inc., Minneapolis, Minnesota) catheter tip consists of a 4.1 mm distal electrode comprised of platinum:iridium (Pt:Ir), chemical vapour deposit (CVD) diamond network, 6 external thermocouples (TCs) and 6 saline irrigation ports. The CVD network allows thermal energy transfer that is 200–400 times faster than achieved with other catheter systems. The catheter provides temperature control to maintain 60 °C at the tissue surface with real-time power modulation, as shown in the representative graphs of power, temperature and impedance change over the course of a single 60 s ablation lesion

Traditional non-irrigated ablation catheters can have deleterious effects because of overheating, such as char and thrombus formation which is associated with an increased risk of thromboembolic complications [4, 5]. This prompted the development of external irrigation catheters which have reduced the frequency of complications associated with overheating, but at the cost of reduced acuity of thermal feedback from the catheter tip, resulting in challenges with titrating energy during ablation [6]. As a result, traditional irrigated catheters are operated in a ‘power control mode’, in order to deliver RF energy. To address these limitations, the DiamondTemp ablation (DTA) catheter (Medtronic, Inc., Minneapolis, MN) was designed to re-establish accurate measurement of tissue temperature. This is accomplished via 6 externally located thermocouples and a chemical vapour deposit (CVD) diamond network to shunt heat from the catheter tip housing 6 saline irrigation ports (Fig. 1a). The CVD network allows thermal energy transfer that is 200–400 times faster than achieved with other catheter systems [7]. The catheter delivers high power RF energy up to 50 W in a ‘temperature control mode’ with real-time power modulation where power delivery is based on the highest thermocouple temperature reading every 20 ms (Fig. 1b).

Preclinical data from computational modelling, thermochromic gel and porcine thigh preparation experiments have guided temperature controlled-ablation parameters [8]. In this study, we aim to demonstrate the feasibility of using the DTA catheter system for VT ablation in patients with ischaemic cardiomyopathy (ICM).

## Methods

This prospective, single-centre study recruited patients who underwent CA for VT with ICM between January and April 2021 at the University Hospital Coventry & Warwickshire (UHCW) NHS Trust.

### Study population

Adult patients (≥ 18 years) with an implantable cardiac defibrillator (ICD) and clinical indications for VT ablation including symptomatic VT despite optimised medical therapy, three or more episodes of VT within 24 h, at least 3 episodes of VT requiring anti-tachycardia pacing (ATP), or at least one appropriate ICD shock were included. All patients provided written consent prior to the procedure. Approval for the study was provided by our Local Audit and Research Department. The study applied the principles of the Declaration of Helsinki.

### Study protocol

All cases were first time procedures carried out according to the UHCW VT ablation workflow as previously described, and all AADs including amiodarone were discontinued for at least 5 days prior to ablation if the stability of arrhythmia allowed it [9]. In brief, the Ensite Precision Cardiac Mapping System (Abbott Medical, Inc., Minneapolis, MN) was used to construct maps with the Advisor HD Grid Mapping catheter (Abbott Medical, Inc., Minneapolis, MN). The DTA catheter (Medtronic, Inc., Minneapolis, MN) was used for ablation. The DTA generator is a temperature-controlled closed-loop system that modulates power to reach and sustain a programmable tip-tissue temperature (default at 60 °C) and an irrigation pump that operates at 8 ml/min while RF energy is delivered. The default DTA parameters were to achieve a target temperature of 60 °C for a maximum of 60 s with initial power ramped to 50 W per lesion. The ablation duration was operator dependent with termination if bipolar electrogram (EGM) reduction or excessive impedance reduction of more than 20 Ω was observed. Metrics recorded for each ablation lesion included power, impedance and temperature from all 6 thermocouples collected at a rate of 1 Hz.

Substrate map data collected for each case included pre- and post-ablation bipolar and unipolar total scar area (TSA), borderzone area (BZA), dense scar area (DSA), late potential area (LPA) and mean bipolar and unipolar voltages of the ablation target area (ATA). Regular bipolar voltage definitions were used to describe preserved myocardial voltage (> 1.5 mV) and dense scar (< 0.5 mV) with LPs identified as isolated component ≥ 20 ms after the end of surface QRS with a detection sensitivity of 0.15 mV [10]. Substrate map areas were measured offline post-procedure using the Ensite Precision Cardiac Mapping System (Abbott Medical, Inc., Minneapolis, MN) surface planimetry tool.

We used programmed electric stimulation (PES) from the right ventricular apex and outflow tract with 2 different drive cycle lengths (600 and 400 ms) and introduction of up to 3 extrastimuli until a ventricular effective refractory period or a coupling interval of 200 ms was reached, without the use of isoproterenol. Clinical VT was determined from the available 12-lead ECG or VT cycle length in the ICD memory. All other monomorphic VTs including polymorphic VT were deemed non-clinical. Non-clinical monomorphic and haemodynamically stable VTs inducible during the procedure were also targeted for ablation. Complete elimination of all clinical and non-clinical monomorphic VTs was defined as complete success. Elimination of the clinical VT only was defined as partial success. Re-induction of clinical VT despite ablation was defined as procedural failure. Major procedure-related complications were defined as those necessitating additional interventions and leading to prolonged hospitalisation.

Post-procedure, ICD programming was left unchanged to facilitate analysis of individual response to ablation, with follow-up for a minimum of 6 months post-ablation. Post-procedure all patients remained on the previously ineffective anti-arrhythmic drug therapy that was administered for at least 6 months prior to ablation. ICD data for the total number of device detected VT episodes, number of device treated VT episodes, VT tachycardia cycle length, number of ATP episodes and number of shocks were collected during the follow-up period and compared to the pre-ablation 6 month period.

### Statistical analysis

Continuous variables are expressed as mean ± SD or median ± IQR if necessary. Statistical significance was assessed using the unpaired Student’s t-test or Mann–Whitney test if necessary. Categorical variables, expressed as numbers or percentages, were analysed using the chi-square test, Fisher’s exact test or McNemar test for paired comparison. Univariate analysis of variables was performed. Cumulative event rates were calculated according to the Kaplan–Meier method. Hazard ratios with corresponding 95% confidence intervals (CIs) are presented. *p* Value < 0.05 defined statistical significance. Statistical analysis was performed using MedCalc and Statview 5.0 statistical software.

## Results

### Patient and procedural characteristics

In total, 10 patients (mean age 73.4 years (range 64–84)) were recruited over 3 months (Table 1). The mean LVEF was 28.6 ± 8.4%, 4 patients were classified as high risk and 6 as intermediate risk using the PAINESD score, with no patient requiring haemodynamic support for the procedure [11]. All patients were established on optimal AAD therapy. The procedural characteristics are summarised in Table 1. In all cases, clinical VT was inducible with an average of 2.1 conduction channels identified per case with the described mapping strategies [9].

**Table 1:** Baseline and procedural characteristics

Age at ablation, years	73.4 ± 6.3
Female sex, *n* (%)	1 (10)
Ethnic Background	
Caucasian, *n* South Asian, *n*	7 (70) 3 (30)
Creatinine, μmol/L	119.8 ± 34.3
Aetiology	
Ischaemic cardiomyopathy, *n*	10 (100)
Past medical history	
AF/flutter, *n* Hypertension, *n* Diabetes, *n* COPD, *n* Cerebrovascular disease, *n*	4 (40) 4 (40) 3 (30) 1 (10) 0 (0)
ICD, *n*	4 (40)
CRT-D, *n*	6 (60)
Pre-ablation ATP, *n*	9 (90)
Pre-ablation shock, *n*	4 (40)
Mean LVEF, %	28.6 ± 8.4
NYHA classification	
I, *n* II, *n* III, *n* IV, *n*	5 (50) 2 (20) 3 (30) 0
Average number of clinical VTs	1.4 ± 0.5
Mean VT cycle length, milliseconds	337.1 ± 73.5
Amiodarone at enrolment, *n*	9 (90)
Β-blocker at enrolment, *n*	10 (100)
PAINESD Score^13^	
Low risk (≤ 8) Intermediate risk (9–14) High risk (≥ 15)	0 6 4
*Average procedure time, min	179.4 ± 52.3
Average fluoroscopy time, min	24.9 ± 8.5
Average substrate map points collected, *n*	26,165 ± 11,384
Average substrate map points used, *n*	2344 ± 917
Clinical VT induced, *n*	10 (100)
Conduction channels identified, *n*	2.1 ± 1.2
Late potentials identified, *n*	10 (100)
Decrementing evoked potentials mapped, *n*	9 (90)
Successful activation map, *n*	10 (100)
Successful pace map, *n*	9 (90)
Successful entrainment map, *n*	3 (30)

^*^Defined as time from vascular access to catheter removal. Values are mean ± SD or *n* (%)

### Ablation lesion data

The DTA lesion characteristics are summarised in Table 2. As the DTA generator provides power, temperature and impedance data every second, there were 2450 ± 1241 data time points analysed per procedure. The average number of ablation lesions per procedure was 51.8 ± 17.7 with an average individual ablation duration of 44.5 ± 11.1 s. The average power and temperature as well as the maximum power and temperature reached per ablation lesion are shown. The time taken to reach the average maximum temperature per ablation of 55.2 ± 2.0 °C was on average 25.8 ± 8.0 s. Similarly, the time taken to reach the average lowest impedance reduction per ablation of 11.2 ± 2.4 Ω was on average 23.0 ± 7.0 s.

**Table 2:** Ablation lesion data

Average number of ablations/procedure, *n*	51.8 ± 17.7
Average total ablation time, min	40.8 ± 20.5
Individual ablation duration, s	44.5 ± 11.1
Average ablation data points, *n*	2450 ± 1241
Average power delivered, Watts	47.8 ± 1.2
Maximum power delivered, Watts	51.8 ± 1.3
Average temperature, °C	49.8 ± 2.0
Maximum temperature reached, °C	55.2 ± 2.0
Time to maximum temperature, s	25.8 ± 8.0
Average number of ablations with temperature > 55 °C, *n* (%)	25.7 ± 8.2 (52 ± 14)
Average ablation start impedance, Ω	97.9 ± 19.3
Average ablation lowest impedance, Ω	86.6 ± 18.4
Average ablation drop in impedance, Ω	11.2 ± 2.4
Time to lowest impedance, s	23.0 ± 7.0
Maximum temperature sensing, %	
TCL1 TCL2 TCL3 TCL4 TCL5 TCL6	22.0 ± 14.1 25.2 ± 19.3 16.8 ± 11.1 9.6 ± 4.9 16.7 ± 11.1 9.7 ± 6.6
Average irrigation fluid infusion, ml	435.3 ± 215.3

Values are mean ± SD or n (%). *TCL* thermocouple. TCL 1–3 = distal; TCL 4–6 = shaft

### Substrate map analysis

**Fig. 2:**
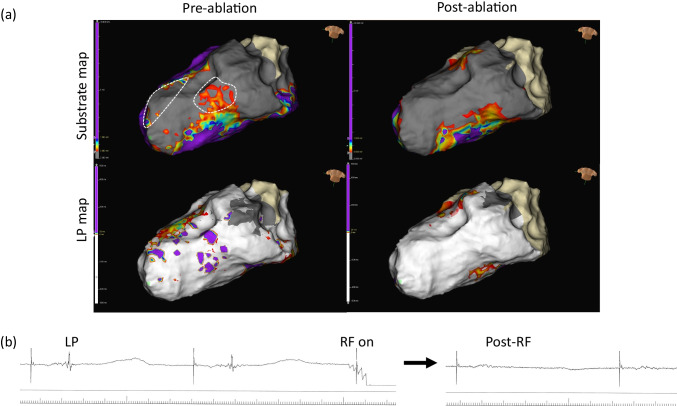
Representative case. Substrate and LP maps are shown in **a** representative case pre- and post-ablation, using standard LV bipolar voltage criteria, with the ATA highlighted with the dashed white line. Post-ablation, the BZA has been effectively reduced from 7.5 to 3.2 cm^2^ with a corresponding increase in the DSA. The pre-ablation LPA (5.8 cm^2^) has been completely eliminated. The mean bipolar voltage of the ATA pre-ablation was 0.43 mV and effectively reduced to 0.36 mV post-ablation. **b** Electrogram amplitude attenuation of the bipolar LP signal from the DTA distal high-resolution electrode is shown (LP = late potential; ATA = ablation target area; BZA = borderzone area; DSA = dense scar area)

Substrate map data analysis is summarised in Table 3. There was a significant reduction in the average bipolar BZA of 4.4 cm^2^/12% (*p* = 0.026) and bipolar LPA of 3.5 cm^2^/83% (*p* = 0.0449) post-ablation. Figure 2a shows representative substrate and LP maps pre- and post-ablation. There was a significant reduction in the mean bipolar (0.14 mV (*p* = 0.0007)) and unipolar (0.59 mV (*p* = 0.0072)) voltages of the ATA post-ablation with Fig. 2b highlighting the reduction in signal attenuation from the DTA catheter. Complete procedural success was achieved in 9 procedures. No post-ablation stimulation protocol was performed at the end of 1 procedure due to a steam pop occurring after 48 min of ablation resulting in pericardial tamponade requiring drainage.

**Table 3:** Substrate map data

	Pre-ablation	Post-ablation	*p* Value
Average bipolar TSA, cm^2^	42.0 ± 26.4	41.2 ± 24.2	ns
Average bipolar DSA, cm^2^	31.4 ± 23.4	36.0 ± 22.2	0.0113
Average bipolar BZA, cm^2^	10.7 ± 5.8	6.2 ± 4.2	0.0255
% Bipolar BZA of TSA	28 ± 11	16 ± 11	0.0260
Average unipolar TSA, cm^2^	44.1 ± 25.5	42.0 ± 20.8	ns
Average unipolar DSA, cm^2^	39.7 ± 25.3	39.5 ± 22.1	ns
Average unipolar BZA, cm^2^	4.3 ± 2.3	2.6 ± 3.0	ns
% Unipolar BZA of TSA	13 ± 10	9 ± 9	ns
Average bipolar LPA, cm^2^	4.2 ± 4.9	0.7 ± 0.9	0.0449
Average bipolar voltage of ATA, mV	0.44 ± 0.16	0.30 ± 0.15	0.0007
Average unipolar voltage of ATA, mV	3.79 ± 1.19	3.20 ± 1.10	0.0072

Values are mean ± SD

This complication occurred in a 69-year-old male with ICM and an ejection fraction of 33% with a CRT-D device. He had a background history of chronic obstructive airways disease and presented with 4 episodes of ATP and 1 shock for VT with a TCL of 352 ms with a high PAINESD score of 19. The substrate map confirmed inferior-septal scar and the VT activation map identified the VT isthmus extending from the septal borderzone to the inferior-lateral borderzone. In total, there were 59 ablation lesions in this case, with an average individual ablation lesion duration of 49 s, with an average 11.8 Ω impedance drop per lesion. The final ablation lesion resulted in a steam pop where the maximum temperature reached was 60 °C with an impedance reduction of 20 Ω targeting the VT isthmus. This was a 44 s duration lesion which achieved a 15 Ω reduction within 10 s after initiation of index ablation, suggesting excessive catheter-tissue force contact. Pericardial tamponade was confirmed followed by intravenous protamine administration to reverse the unfractionated heparin as the pericardial drain was inserted. Approximately 100 ml of blood was autotransfused and the patient stabilised. The drain was removed the following day and the patient recovered well.

### Follow-up data

**Fig. 3:**
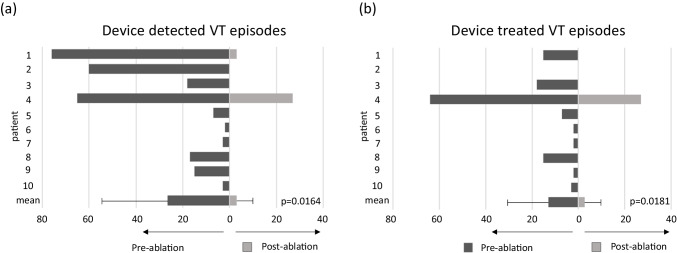
Follow-up data. Histograms showing individual **a** device detected VT episodes and **b** device treated VT episodes within the 6 months pre-ablation period compared to the minimum 6 months post-ablation follow-up. Pre-ablation device detected VT episodes *n* = 266; median 16 [IQR 3–57]. Post-ablation device detected VT episodes *n* = 33; median 0 (*p* = 0.0164). Pre-ablation device treated VT episodes *n* = 128 median 5 [IQR 2–15]. Post-ablation device treated VT episodes *n* = 30; median 0 (*p* = 0.0181). Values are mean ± SD.

Figure 3 highlights the individual reductions in total device detected VT and device treated VT episodes. All patients underwent a minimum of 6 months follow-up with a mean of 214 ± 33 days. In the 6 months prior to ablation, there were a total of 266 device detected VT episodes, with a mean VT cycle length of 337 ± 71 ms, of which 128 were device treated VT episodes (120 ATP; 8 shocks). At final follow-up, a total of 33 device detected VT episodes were observed with a mean VT cycle length of 410 ± 10 ms, of which 30 were device treated VT episodes (27 ATP; 3 shocks). A significant 88% reduction in total VT episodes (*n* = 266 median 16 [IQR 3–57] to *n* = 33 median 0; *p* = 0.0164) and 77% reduction in ICD therapies (*n* = 128 median 5 [IQR 2–15] to *n* = 30 median 0; *p* = 0.0181) were observed. No deaths occurred in the study cohort.

## Discussion

This single-centre study reports the first in-human experience of temperature-guided irrigated RF ablation for VT ablation using the DTA system. The novel design of the DTA catheter with an array of externalised thermocouples situated directly at the tip-tissue interface permits reintroduction of temperature-controlled irrigated RF ablation. The main findings of this study are that temperature-controlled irrigated RF ablation (1) delivers adequate power titration to achieve and maintain target tissue temperature and resultant tissue impedance reductions, (2) is effective in eliminating the VT substrate, and (3) results in a significant reduction in intermediate-term VT burden and ICD therapies.

Effective ablation lesions are produced when irreversible tissue damage occurs; at tissue temperatures of > 50 °C [12]. The DTA system operates in a ‘temperature control mode’, with a constant feedback loop between the catheter thermocouples and the generator that optimises power output every 20 ms. This ensures that effective lesions are created without excessive catheter tip temperatures. We were able to achieve an average temperature of 49.8 °C with an average maximum temperature of 55.2 °C per ablation lesion. The time taken to achieve this maximum temperature was on average 25.8 s post-RF delivery. Interestingly, we observed the lowest impedance reductions at a similar time point of 23 s.

The efficacy and safety of the DTA system has been reported for atrial fibrillation ablation [5, 7]. The DIAMOND-AF study recently reported the noninferiority of the DTA system to a force-sensing ablation system for pulmonary vein isolation [5]. The ablation recommendations in DIAMOND-AF were to achieve a target temperature of 60 °C, and the average individual ablation duration was 14.7 ± 5.3 s. Based on preclinical data from computational modelling, thermochromic gel and porcine thigh preparation experiments, we proposed 60 s lesions that were guided by bipolar EGM and impedance changes [8].

### Independent relationship between Impedance and temperature change in lesion formation

Impedance reduction is routinely monitored in clinical practice to assess RF ablation delivery having been shown to be an important indicator of successful lesion formation [13–15]. Previous studies have demonstrated the correlation between impedance reduction and lesion dimension with impedance reductions of 10 Ω regarded as a marker of adequate lesion formation [14, 15]. The initial impedance reduction during RF ablation is larger when greater catheter contact is achieved [14]. Indeed, it has been reported that impedance reductions of ≥ 10 Ω cannot be achieved with contact forces less than 5 g under any power setting [16]. Preclinical data using the DTA system in a porcine thigh preparation shows a linear relationship between impedance reduction, duration of RF delivery and lesion depth [8]. Therefore, despite the non-contact force nature of the DTA catheter, adequate impedance reductions would support effective tissue contact.

**Fig. 4:**
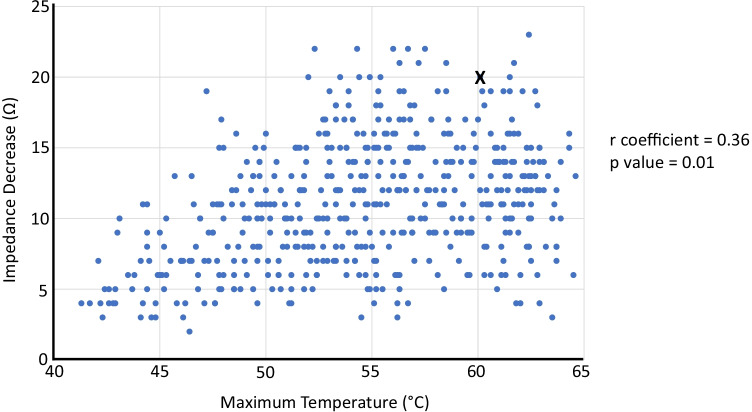
Temperature versus Impedance. Scatter plot of all 518 ablation lesions demonstrating a poor correlation between maximum temperature and impedance decrease (r coefficient = 0.36; *p* value = 0.01). Lesion X identifies ablation lesion resulting in steam pop and pericardial tamponade

Through design, the DTA catheter is expected to provide rapid thermal diffusivity and temperature feedback to allow for high power energy delivery. We observed a single steam pop that resulted in pericardial tamponade suggesting insufficient thermal feedback and power titration. Figure 4 represents a scatter plot of all 518 ablation lesions delivered within this study and 322 (62%) lesions resulted in impedance reductions greater than 10 Ω. As termination of RF delivery was operator guided and not an automated process, 18 (3%) lesions resulted in impedance reductions greater than 20 Ω. Interestingly, we observed a poor correlation between the maximum temperature reached and the impedance reduction per ablation lesion. This observation suggests that the catheter tip-tissue interface temperature alone may not be a solely reliable marker for safe and effective DTA lesion delivery. Indeed, the increased thickness of ventricular tissue, compared to atrial tissue, may facilitate increased temperatures farther from the catheter tip that cannot be detected by the externalised thermocouples.

Computational modelling studies applying the DTA system have shown that tip-tissue interface temperatures ranging from 52.7 to 60.3 °C will result in maximum internal tissue temperatures ranging from 82.8 to 94.0 °C [8]. Furthermore, applying DTA lesions with a target temperature of 60 °C and maximum power of 50 W to a thermochromic gel preparation achieved tip-tissue interface temperatures of up to 64 °C with no application producing internal tissue temperatures greater than 80 °C [8]. These lesion applications were limited to 15 s, and the longer application duration delivered with lesion X may have resulted in excessive tissue temperature despite an optimal tip-tissue interface temperature. The mechanism for this may have been excessive resistive heating which manifested as an excessive reduction in impedance.

We therefore propose that with temperature-guided irrigated RF ablation for VT ablation, both maximum temperature and impedance changes, are utilised to tailor the ablation duration. Our ablation protocol was to deliver 60 s ventricular lesions that were guided by bipolar EGM and impedance changes, and based on these parameters, the average individual ablation duration was 44.5 ± 11.1 s. This ablation duration is significantly longer than the 14.7 ± 5.3 s average individual ablation duration reported for atrial ablation in the DIAMOND-AF study where ablation termination guidance was 3 to 5 s beyond electrogram amplitude attenuation of 75 to 80% [5]. Based on the current commercially available DTA generator and the data that is provided in real-time, our data study would support individual ventricular ablation durations limited to impedance reductions of no greater than 15 Ω, as was the case in 84% of lesions in this study.

### Importance of direct temperature sensing from catheter tip thermocouples

The importance of direct temperature sensing from both the shaft thermocouples and distal tip thermocouples is reflected in our data. Of the total maximum temperatures recorded for all lesions, only 64% were from the 3 distal tip thermocouples, while the remainder were detected on the externalised shaft thermocouples. We observed a poor correlation between catheter orientation to tissue in predicting which thermocouple would detect the highest temperature. In case 8, the target for ablation was the apical and septal mid-LV and 88% of the maximum temperatures detected were on the distal thermocouples. In case 5, where the septal and apical LV was targeted with a similar catheter orientation perpendicular to the myocardium, only 68% of the maximum temperatures detected were on the distal thermocouples. However, in case 1 where the ablation target area was the inferior mid-LV with a similar catheter orientation perpendicular to the tissue, only 54% of the maximum temperatures detected were on the distal thermocouples. On the contrary in cases 2 and 10, where the catheter tip was parallel to the myocardium with the ablation target area at the lateral basal LV and inferoseptal basal LV, 56% and 68% of the maximum temperatures detected were on the distal thermocouples, respectively.

A comparison of the time required to reach maximum temperature in cases (*n* = 6) of a good correlation of catheter orientation to the contacting/sensing thermocouples (301 individual ablation lesions) versus cases (*n* = 4) with a poor correlation of catheter orientation to the contacting/sensing thermocouples (217 individual ablation lesions) was performed. The time taken to reach maximum temperature was significantly longer in the cases of poor catheter orientation correlation to the contacting/sensing thermocouples (30.2 ± 19.1 s vs 24.8 ± 17.7 s; *p* = 0.001) suggesting the poor correlation may be due to reduced or angulated catheter contact or the tip was embedded within trabeculated ventricular structures. However, this conclusion is speculative due to the absence of real-time intracardiac imaging, contact force or force vector data to identify which thermocouple(s) had the real, tightest catheter-tissue contact. These observations do however highlight the importance of the externalised shaft thermocouples in contributing to power modulation.

### Safety

All patients included in this study were classified as intermediate or high risk using the PAINESD score with significant co-morbidities [11]. Apart from one pericardial tamponade which required a pericardial drain for 24 h, there were no other steam pops or significant post-procedural complications. The DTA system delivers high power RF energy; however, because of the catheter tip CVD diamond network and resulting rapid heat dissolution, the irrigation flow rate remains low at 8 ml/min. The average ablation fluid irrigation per case was 435.3 ml which would represent a 3- to fourfold reduction compared to standard irrigated RF catheters such as the TactiCath (Abbott Medical, Inc., Minneapolis, MN) or Smart Touch (Biosense Webster, Inc.) ablation catheters [9]. Whether this reduction in fluid volume would translate into reduced post-procedural decompensated heart failure is yet to be shown.

### Efficacy

The efficacy of the DTA lesions was reflected in the substrate and LP map analyses pre- and post- ablation along with the post-procedural VT inducibility testing. We demonstrated a 12% reduction in average bipolar BZA with a corresponding 15% increase in bipolar DSA post-ablation. Furthermore, the LPA was significantly reduced by 83%. High density maps were generated using the Advisor HD Grid Mapping catheter, which has been associated with improved long-term post-procedural outcomes [7, 9]. Accordingly, we demonstrated a significant reduction in the mean bipolar and unipolar voltages of the ATA consistent with effective substrate modification. These findings were further supported by non-inducibility of VT during end-point testing. The effectiveness in substrate elimination and VT non-inducibility post-procedure translated into positive clinical outcomes over the follow-up period of 6–10 months with a significant reduction in total VT episodes and ICD therapies.

### Study limitations

This study was a feasibility study designed to explore the ability of the DTA system to deliver effective temperature-controlled irrigated RF ablation for VT ablation. As such, the current study has a small sample size, included patients with ICM only, and follow-up was limited to a minimum of 6 months per patient. Furthermore, post-procedure ICD programming was left unchanged to facilitate analysis of individual responses to ablation; however, this may have resulted in the relatively common post-ablation observation of under-detected slower VTs. The DTA system was not compared to other ablation catheters that can also deliver high power and additional contact force data. Larger comparative studies with formal hypothesis and statistical power calculations are required to definitively investigate the safety and efficacy of the DTA system. Future randomised controlled trials are required to compare the performance of the DTA system against conventional RF ablation catheters. Studies are also required to assess the effectiveness and safety of the DTA system in patients with a non-ischaemic VT substrate.

## Conclusions

This is the first-in-human study to report the ablation characteristics, efficacy in substrate elimination and intermediate-term success rates of temperature-controlled irrigated RF for VT ablation. Larger trials are ongoing to confirm safety and long-term effectiveness of this novel ablation technology. The DTA system delivered power-modulated, temperature-controlled ablation lesions with associated impedance reductions that resulted in successful substrate elimination and complete elimination of all clinical and non-clinical VTs. These findings were substantiated by the intermediate follow-up outcomes with a reduction in overall VT burden and ICD therapies.
